# The role of 'confounding by indication' in assessing the effect of quality of care on disease outcomes in general practice: results of a case-control study

**DOI:** 10.1186/1472-6963-5-10

**Published:** 2005-01-27

**Authors:** Johan S de Koning, Niek S Klazinga, Peter J Koudstaal, Ad Prins, Gerard JJM Borsboom, Johan P Mackenbach

**Affiliations:** 1 Department of Public Health, Erasmus MC, University Medical Centre Rotterdam, the Netherlands; 2 Department of Social Medicine, Academic Medical Centre, University of Amsterdam, the Netherlands; 3 Department of Neurology, Erasmus MC, University Medical Centre Rotterdam, the Netherlands; 4 Department of General Practice, Erasmus MC, University Medical Centre Rotterdam, the Netherlands

## Abstract

**Background:**

In quality of care research, limited information is found on the relationship between quality of care and disease outcomes. This case-control study was conducted with the aim to assess the effect of guideline adherence for stroke prevention on the occurrence of stroke in general practice. We report on the problems related to a variant of confounding by indication, that may be common in quality of care studies.

**Methods:**

Stroke patients (cases) and controls were recruited from the general practitioner's (GP) patient register, and an expert panel assessed the quality of care of cases and controls using guideline-based review criteria.

**Results:**

A total of 86 patients was assessed. Compared to patients without shortcomings in preventive care, patients who received sub-optimal care appeared to have a lower risk of experiencing a stroke (OR 0.60; 95% CI 0.24 to 1.53). This result was partly explained by the presence of risk factors (6.1 per cases, 4.4 per control), as reflected by the finding that the OR came much closer to 1.00 after adjustment for the number of risk factors (OR 0.82; 95% CI 0.29 to 2.30). Patients with more risk factors for stroke had a lower risk of sub-optimal care (OR for the number of risk factors present 0.76; 95% CI 0.61 to 0.94). This finding represents a variant of 'confounding by indication', which could not be fully adjusted for due to incomplete information on risk factors for stroke.

**Conclusions:**

At present, inaccurate recording of patient and risk factor information by GPs seriously limits the potential use of a case-control method to assess the effect of guideline adherence on disease outcome in general practice. We conclude that studies on the effect of quality of care on disease outcomes, like other observational studies of intended treatment effect, should be designed and performed such that confounding by indication is minimized.

## Background

There is a long tradition of studying at population level the quality of medical care provided to patients who died from conditions amenable to medical intervention. This type of study (so called 'in-depth' or 'audit' study), aims to identify deficiencies in medical care that may have contributed to death. It was first systematically carried out on maternal death, and later on other causes of avoidable death [1-4]. This method can be applied to other potentially avoidable conditions, e.g. those that could be avoided by appropriate preventive care. The general approach is to document in detail the process of care provided to a single patient preceding the occurrence of an adverse event, followed by an assessment of the quality of care by an expert panel, either with or without the use of explicit criteria [5].

An important limitation of this type of study, without control subjects, is its inability to fully establish a causal relationship between identified deficiencies in care and the adverse outcome, and to determine to what extent identified deficiencies are associated to the occurrence of such an event. Identified deficiencies in care are expected to indicate only to a certain extent an increase in risk of an adverse health outcome, while the probability of having an adverse outcome can be calculated only if we compare the care provided to patients who suffered an adverse outcome with that of patients who did not suffer such an event. For this reason it has been proposed to perform a case-control study with patients with an adverse event as 'cases' and a comparable group of patients without an adverse outcome as 'controls' [6].

We performed a case-control study with the aim to assess the effect of guideline adherence for stroke prevention on the occurrence of stroke in general practice. Unfortunately, we encountered various obstacles in the design and conduct of this study, in particular related to the recruitment of cases and controls, in availability of information on the care delivery process in the GP's data registration system, and in controlling for differences other than differences in the quality of care. The aim of this paper is to highlight the problems related to a variant of confounding by indication, that may be common in quality of care studies.

Observational studies of intended treatment effects are particularly prone to 'confounding by indication', and can produce misleading estimates on either, or both, the size and direction of treatment effects [7,8]. Confounding by indication refers to an extraneous determinant of the outcome parameter that is present if a perceived high risk or poor prognosis is an indication for intervention. This means that differences in care, for example, between cases and controls may partly originate from differences in indication for medical intervention such as the presence of risk factors for particular health problems. The latter has frequently been reported in studies evaluating the efficacy of pharmaceutical interventions [9,10], screening tests [11], and vaccines [12]. We hypothesise that this may not only apply to indications for medical intervention but also for guideline adherence and quality of care. In comparing retrospectively the quality of care between patients with and without a stroke, stroke patients may have received more preventive care because more indications for preventive interventions were present. Because differences in indications for preventive intervention correspond with the probability of an adverse outcome (more indications will be associated with a higher risk of an adverse outcome), when comparing care between cases and controls it is necessary to control for these differences. If one omits to control for confounding by indication, it is expected that more and probably better care, correlates with a higher risk of stroke. In quality of care research, there is, as yet, little information regarding the role of confounding by indication in studies that investigate the effect of quality of care on disease outcomes.

## Methods

### Sample

From the Dutch national GP register, a random sample was taken of 58 GPs working in Rotterdam and the surrounding region. The study was restricted to patients with a first-ever stroke meeting the following criteria for inclusion: (a) diagnosis of intracerebral haemorrhage or infarction according to the World Health Organization (WHO) definition of stroke [13], (b) age between 39–80 year, (c) occurrence of stroke in the period 1996–1997, (d) stroke caused by cardiovascular disease (CVD) and not by trauma, infection or malignancy, (e) presence of hypertension, (f) GP of the patient practising in the southern part of Rotterdam or surrounding region, (g) patient registered with local GP for not less than two years, and (h) patient not living in a nursing home during the two years period prior to stroke. Cases and controls were selected from the GPs' patient register, using health outcome (stroke) and risk factor (e.g. hypertension) entries. For each case, two controls were randomly selected and matched with the cases in terms of overall distribution on sex, age, and hypertension (most important risk factor for stroke). Cases and controls were not matched on the same GP.

### Data collection

In a pilot study among 32 GPs, the quality of care measurement instruments (audit procedure and questionnaire) were tested. GPs participating in the pilot study did not participate in this study. Data on the process of care, two years prior to the occurrence of stroke (for controls from January 1995 to January 1997), were collected by means of structured face-to-face interviews with the GP, using separate questionnaires for each stroke patient. GPs were interviewed between March and October 1999. At the time of interview, GPs used either hand-written or electronic patient records to retrieve patient information. In case information was not available in the patient's record, information was drawn from the GP's memory. For each question, the type of data source was registered. The questionnaire comprised questions related to patient characteristics and family and medical history of CVD and risk factors, and the detection and treatment of cardiovascular risk factors such as hypertension, diabetes mellitus, transient ischemic attack (TIA) and cardiac failure. Similarly, data were collected on lifestyle-related risk factors such as smoking status, overweight, and excessive alcohol intake.

### Expert panel and assessment method

The quality of preventive care and its potential to prevent stroke was assessed and valued by a six-member panel of experts. The panellists (three neurologists and three GPs) were selected on the basis of their clinical expertise with respect to stroke prevention, experience in quality of care evaluation, academic or non-academic background and professional discipline. Six practice guidelines relevant to stroke prevention (hypertension, diabetes mellitus, TIA, peripheral vascular disease, cardiac failure and angina pectoris) were selected by the panel [14]. These guidelines, based on scientific evidence, broad consensus, and clinical evidence, are developed and implemented by the Dutch College of General Practitioners as part of a national guideline program operational since 1987 [15]. From each guideline, the panellists identified specific elements of care and systematically converted these into review criteria (n = 65), allowing detailed measurement of GP's adherence [16]. All these criteria were all used to construct the patient questionnaire.

In a two-round evaluation, with a final plenary round, cases were assessed by the panellists (panellists were divided in sub-panels). Each sub-panel assessed a specific number of cases. Based on identified elements of sub-optimal care and seriousness of shortcoming in terms of 'minor' and 'major', the panellists allocated grades on a scale of 0 to 3 (Table 1).

**Table 1:** Grades of (sub)optimal care given by the expert panel (in both groups allpatients are hypertensive)

			*Cases*		*Controls*	
			
			*n*	*%*	*n*	*%*
Grading:	0	No sub-optimal factors have been identified	12	43	18	31
	1	Sub-optimal factor(s) have been identified, but are unlikely to be related to the occurrence of stroke in this patient	8	29	18	31
	2	Sub-optimal factor(s) have been identified, and possibly have failed to prevent the stroke in this patient	4	14	18	31
	3	Sub-optimal factor(s) have been identified, and are likely to have failed to prevent the stroke in this patient	4	14	4	7
Sub-optimal care		Grading 1, 2, 3	16	57	40	69

Total		Grading 0, 1, 2, 3	28	100	58	100

The two-round process was focussed on detecting consensus among the panellists (providing the same grade), and no attempt was made to force the panellists to consensus. The intersubpanel agreement was k = 0.63 (overall agreement on assigned grades between sub-panels was 74%). A detailed description of the assessment method is provided elsewhere [17].

### Analysis

Analysis of the data was done by using simple cross-tabulations, and by using logistic regression analysis to model the chance of getting a stroke as a function of the presence of sub-optimal care (as ascertained by the panel), age and sex, and risk factors for stroke.

## Results

### GP Participation and recruitment of cases / controls

The rate of participation was 62% (36 GPs). The main reason for GPs not to participate in the study was lack of time and interest (68%). Participating and non-participating GPs did not differ significantly in age, practice type, and date of qualification. Ninety-two percent of the GPs used electronic GP information systems. Among cases and controls there was a nonsignificant difference in mean age, however, cases were slightly older than controls (67 versus 65 years). Initially, before we excluded patients 'without' hypertension, GPs identified and selected 50 cases and 58 controls (1.4 case and 1.6 control per GP). Expected number of cases was 2.5 stroke patients per GP per year [18]. After excluding patients without hypertension, 28 cases and 58 controls with hypertension entered the study.

### Availability of data

Overall, data for verification of the initial diagnosis of stroke, assessment of GPs' guideline adherence, and judgement of the causality of the relationship between non-adherence and the occurrence of stroke could be collected from the patient records. However, information on risk factors such as family history of CVD, body weight (overweight), excessive alcohol intake, and smoking was less easily obtained. Depending on the type of risk factor, in 8–56% of all subjects, information on risk factors was unknown to the GP (8% in patients with overweight, 11% in patients smoking cigarettes, 17% in patients with excessive alcohol consumption, and 56% in patients with a family history of CVD). In 41–58% information was taken from the GP's memory, instead of the patient register.

### Indications for confounding by indication

In 43% of the cases and 31% of the controls, no sub-optimal care could be identified (grade 0), whereas in 57% and 69%, respectively, sub-optimal care was identified (grade 1, 2 or 3). Thus the Odds Ratio for a case to receive sub-optimal care was 0.60 (95%CI 0.24 – 1.53) compared to a control (Table 1). Compared with controls receiving sub-optimal care, the number of shortcomings in care per case receiving sub-optimal care was higher (28/16 = 1.7 versus 41/40 = 1.0) (Table 2). The percentage of shortcomings in hypertensive care, however, was considerably higher among controls (90% versus 57%, respectively). The latter, apparently, correlates with the fact that controls less often have risk factors other than hypertension (next paragraph).

**Table 2:** Guideline-derived elements of care used to indicate shortcomings in care among stroke patients and controls

*Practice guideline*	*Elements of care*	*Cases*	*Controls*
			
Arguments derived from practice guideline: Hypertension	- Detection of hypertension	1	2
	- Confirmation diagnosis hypertension	2	1
	- Pharmacologic therapy (anti-hypert. med)	2	1
	- Follow-up (quarterly)	8	17
	- Follow-up (annually)	3	16
			
Arguments derived from practice guideline: Diabetes mellitus	- Follow-up (quarterly)	4	3
	- Laboratory evaluation	1	0
	- Referral to eye specialist	1	0
			
Arguments derived from practice guideline: TIA	- Treatment (therapy and follow-up after TIA)	1	1
			
Arguments derived from more than one practice Guideline	- Advice to quit smoking	2	0
	- Dietary advice (overweight)	1	0
	- Evaluation of cardiovascular risk profile	2	0
			

Total number of shortcomings		28	41
Total number of patients with shortcomings		16	40

TIA, Transient Ischemic Attack

Note: each patient could have more than one element of sub-optimal care

**Figure 1:**
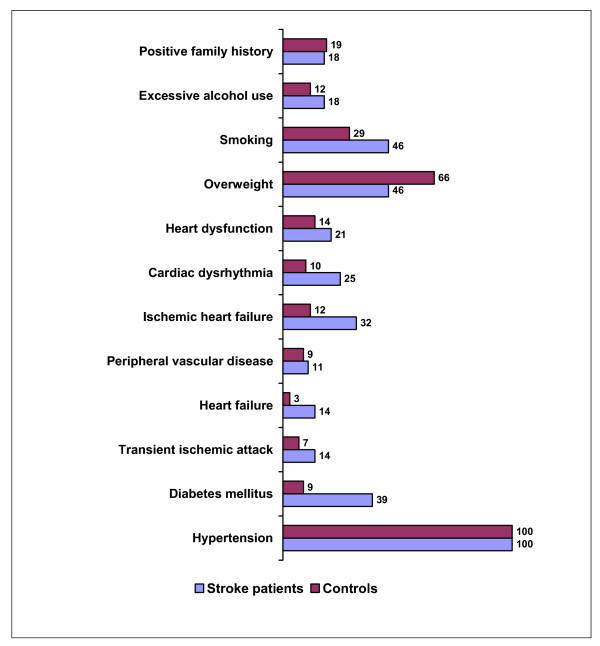
Risk factor distribution. Prevalence (%) of risk factors for stroke among stroke patients (n = 28) and controls (n = 58). Total number of risk factors among stroke patients is 172, and among controls 277. Mean number of risk factors per case is 6.1, and for controls 4.4. This relationship is statistically borderline significant (p = 0.096), and could be an explanation for the somehow surprising result found earlier, that is, that cases receive sub-optimal care less often than controls.

The mean number of risk factors among cases (6.1 per patient) was higher than among controls (4.4 per patient) (Figure 1). Multivariate logistic regression indicates that cases receiving sub-optimal care (grade 1, 2, or 3) have a lower risk of stroke (crude OR 0.60) (Table 3). If adjusted for sex and age distribution, the odds ratio does not change significantly (adjusted OR 0.64). Subsequently, in an attempt to investigate the possible role of confounding by indication we adjusted for risk factor prevalence. Indeed, with an adjusted OR of 0.82 (95% CI 0.29–2.30), it seems that risk factor prevalence to some extent explains why patients receiving sub-optimal care have a lower risk of stroke.

**Table 3:** Relationship between quality of care and the occurrence of stroke(Odds Ratio and 95% CI)

	MODEL 1	MODEL 2	MODEL 3
			
Care:			
Optimal	1.00 (ref.)	1.00 (ref.)	1.00 (ref.)
Sub-optimal	0.60 (0.24–1.53)	0.64 (0.25–1.65)	0.82 (0.29–2.30)
			
Sex:			
Male		1.00 (ref.)	1.00 (ref.)
Female		0.90 (0.36–2.30)	0.61 (0.22–1.72)
			
Age:		1.03 (0.98–1.08)	1.03 (0.98–1.08)
			
Risk factors:			0.76 (0.61–0.94)

Note: to control for risk factors, the number of risk factors per patient were included in the regression model.

Patients with a higher number of risk factors for stroke, indeed have a lower risk of sub-optimal care (OR for the number of risk factors present 0.76; 95% CI 0.61–0.94). As expected, higher numbers of risk factors per patient also increases the risk of stroke (OR for the number of risk factors present 1.34; 95% CI 1.10–1.62).

## Discussion

This study demonstrated confounding by indication in a case-control study analysing the association between guideline adherence and the occurrence of stroke in general practice. It also provided insight into the possibilities for controlling for this confounding bias. We learned that, at present, difficulties in patient recruitment and data retrieval seriously limit the potential use of a case-control method to assess the relationship between guideline adherence for stroke prevention and stroke in general practice.

We found that in specific domains data were incomplete and not readily available in the patient records. As a consequence, in many cases GPs were unable to identify stroke patients from their patient register, which most likely introduced under-reporting of stroke patients. As compared to national frequencies (2.5 stroke patients per GP per year) [18], GPs participating in our study identified less stroke patients, 1.7 stroke patient per GP. The same applies to information on patients' family history of CVD and lifestyle-related risk factors, which was inaccurate and in many cases not available in the patient's register. The latter finding is consistent with previous work on the accuracy of information on CVD risk factors in GPs' patient records [19,20], indicating that data from GP's record on lifestyle-related risk factors of CVD are frequently incomplete or absent. Incomplete information on risk factors for stroke is a serious threat to the validity of the results of case-control studies investigating the relationship between process of care and health care outcome. It complicates evaluation of GP's adherence to recommended guidelines, and makes it difficult, if not impossible, to control for confounding by indication. Apart from that, information on risk factors that was available in the patient records is presumably not 100% valid.

Strong indications for the existence of confounding by indication were found, albeit different from how it is usually described in literature. Confounding by indication, which is conceived as a substantial problem in observational studies of treatment efficacy, usually refers to a situation in which patients who are more in need both receive more care have a higher risk of adverse health outcome [8]. In our study, we show that confounding by indication can also cause patients with an adverse health outcome (stroke) to appear to receive better quality of care.

A more detailed analysis showed, similar to results found in previous studies, that this result partly emanates from a higher prevalence of risk factors for stroke among patients suffering stroke at a later stage in life, which not only increases the risk of stroke but also GPs' compliance to guidelines. We hypothesize that, on average, patients with more risk factors for stroke receive more attention or visit their GP more frequently, which in turn facilitates guideline adherence (e.g. compliance to quarterly follow-up of treated hypertensive patients) and at the same time results in better quality of care. Controlling for (recorded) risk factors reduced the counter-intuitive result by approximately one half, and we hypothesise that incomplete registration of risk factors for stroke explains why the risk of stroke in stroke-prone or high-risk patients associated with sub-optimal care remained below 1.00, even after controlling for risk factors. We hope that our paper draws the attention of quality of care researchers to this variant of confounding by indication, that may lead to biased associations between process measures of quality of care and care outcomes.

## Conclusions

This study shows that, at present, difficulties in patient recruitment and data retrieval seriously limit the potential use of a case-control method to assess the relationship between guideline adherence for stroke prevention and stroke in general practice. It demonstrates the role of confounding by indication, causing patients with an adverse health outcome to appear to receive better quality of care.

## Competing interests

The author(s) declare that they have no competing interests

## Authors' contributions

JK, NK, PK, AP and JM conceived and designed the study. Analyses were performed by JK and GB. All authors contributed to this article and earlier drafts of the manuscript.

## Pre-publication history

The pre-publication history for this paper can be accessed here:


